# Tobacco Smoking in Early Adulthood and Labor Market Performance: The Cardiovascular Risk in Young Finns Study

**DOI:** 10.1093/ntr/ntae296

**Published:** 2025-01-14

**Authors:** Jutta Viinikainen, Petri Böckerman, Christian Hakulinen, Jaana T Kari, Terho Lehtimäki, Katja Pahkala, Jaakko Pehkonen, Jorma Viikari, Olli T Raitakari

**Affiliations:** Jyväskylä University School of Business and Economics, University of Jyväskylä, Jyväskylä, Finland; Jyväskylä University School of Business and Economics, University of Jyväskylä, Jyväskylä, Finland; Labour Institute for Economic Research LABORE, Helsinki, Finland; IZA Institute of Labor Economics, Bonn, Germany; Department of Psychology and Logopedics, University of Helsinki, Helsinki, Finland; Welfare State Research Unit, Department of Healthcare and Social Welfare, Finnish Institute for Health and Welfare, Helsinki, Finland; Jyväskylä University School of Business and Economics, University of Jyväskylä, Jyväskylä, Finland; Faculty of Medicine and Health Technology, Tampere University, Tampere, Finland; Finnish Cardiovascular Research Center, Tampere University, Tampere, Finland; Fimlab Laboratories, Tampere, Finland; Centre for Population Health Research, University of Turku and Turku University Hospital, Turku, Finland; Research Centre of Applied and Preventive Cardiovascular Medicine, University of Turku, Turku, Finland; Paavo Nurmi Centre and Unit for Health and Physical Activity, University of Turku, Turku, Finland; Jyväskylä University School of Business and Economics, University of Jyväskylä, Jyväskylä, Finland; Department of Medicine, University of Turku, Turku, Finland; Division of Medicine, Turku University Hospital, Turku, Finland; Centre for Population Health Research, University of Turku and Turku University Hospital, Turku, Finland; Research Centre of Applied and Preventive Cardiovascular Medicine, University of Turku, Turku, Finland; Department of Clinical Physiology and Nuclear Medicine, Turku University Hospital, Turku, Finland

## Abstract

**Introduction:**

Tobacco smoking has been associated with reduced success in the labor market, potentially due to its negative impact on labor productivity, especially in physically demanding jobs, as it affects physical fitness and performance adversely.

**Methods:**

This prospective study used data from the Cardiovascular Risk in Young Finns Study survey, linked to register information on labor market outcomes and education attainment, to examine the association between tobacco smoking and long-term labor market outcomes (earnings and employment, *N* = 1953). Smoking levels were determined by cigarette pack-years in 2001, as reported in the survey, whereas annual earnings and employment status were tracked from 2001 to 2019.

**Results:**

A one-unit increase in pack-year of smoking was associated with a 1.8% decrease in earnings (95% confidence interval [CI]: −2.6% to −0.9%) and a 0.5% reduction in years employed (95% CI: −0.6% to −0.3%). This association was pronounced among participants with lower education levels. The earnings difference was evident among younger cohorts, whereas a negative correlation with employment was observed most strongly in older cohorts among individuals with lower education.

**Conclusions:**

Our findings suggest that smoking had a negative effect on earnings among the younger generation, particularly among the less well-educated. The finding of greater impacts on years of employment among the older age group, particularly among groups with low education levels, is consistent with the delayed onset of most health impacts, which may particularly affect productivity in physically demanding jobs that are more common among people with less education.

**Implications:**

Adverse consequences of smoking include reduced earnings and labor market participation, particularly among less well-educated groups. Tobacco control advocates should draw attention to these consequences in arguing for effective measures to reduce smoking initiation and increase cessation in order to achieve socially optimal outcomes.

## Introduction

The adverse health effects of tobacco smoking are well-documented.^1^ Smoking elevates the risk of various cancers, respiratory issues, and cardiovascular diseases, with approximately 14% of all deaths in 2019 attributed to smoking.^2^ Despite a declining trend of smoking since the 1990s, its age-standardized prevalence in high-income countries was 18% among females and 27% among males in 2019.^2^

Besides posing health risks, tobacco smoking has been linked to decreased labor market performance, including lower earnings.^3–7^ This may be due to severe health problems caused by smoking, resulting in an inability to work, increased sick absences, and premature retirement. However, not all smokers experience such severe health problems impeding their ability to work. Smoking may also lead to reduced physical fitness and performance, impacting workplace productivity, which may limit earning potential, particularly in physically demanding occupations. Moreover, the stigma surrounding smoking may induce bias and discrimination against smokers.^8^

This study investigated the relationship between smoking combustible cigarettes and labor market outcomes (earnings and employment), thus augmenting previous research in three ways. First, we used longitudinal data and register-based employment records to examine labor market outcomes over 18 years between smokers versus nonsmokers. Second, since the physical demands of the occupation can aggravate the harmful impact of smoking on work performance, we examined whether the results differ by the level of education. Third, we measured tobacco smoking using pack-years, indicating cumulative lifetime cigarette consumption.

## Methods

### Data Sources and the Sample

The Cardiovascular Risk in Young Finns Study (YFS) is a longitudinal study of 3596 participants from urban and rural areas of five Finnish university regions, born between 1962 and 1977, representing six age cohorts.^9^ The YFS data were linked to labor market outcomes from Statistics Finland (the FOLK longitudinal data modules, FOLK) and parental background information from the Longitudinal Population Census (LPC) using personal identifiers. The observation period, starting in 2001, covered participants aged 24—39. For the study subjects’ flowchart, refer to Supplementary Figure S1.

### Measures

The study used the logarithm of annual wage and salary earnings, as well as employment status (1 = employed; 0 otherwise) from FOLK data as outcome variables, focusing on long-term averages from 2001 to 2019, along with additional analyses covering a shorter follow-up period from 2010 to 2019. Long-term averages were preferred due to their stability over short-term measures, which contain more random variation leading to larger standard errors and attenuation of parameter estimates.

We measured smoking using cigarette pack-years from the 2001 YFS survey, capturing cumulative lifetime smoking. Pack-years were calculated by multiplying the average daily cigarettes smoked at the time of the survey by the person’s age minus the age at smoking initiation. For instance, a person with a 10-pack-year history of smoking has smoked one pack daily for 10 years. We focused solely on combustible cigarettes, as they were the predominant form of tobacco consumption in Finland during the study period. Furthermore, pack-years are typically calculated based on combustible cigarette use in the research literature.

Using educational attainment information from FOLK, YFS participants were categorized as highly educated if they had completed the International Standard Classification of Education (ISCED) level 5 or above in 2001, while those with lower qualifications were considered low-educated. Alternatively, we used a three-level categorization of education: comprehensive education (ISCED level 2), intermediate education (ISCED levels 3 or 4), and higher education (ISCED level 5 or above) in 2001.

Sex (FOLK), birth cohort (six indicators, YFS), region of residence in 1980 (four indicators, YFS), and parental background indicator (LPC) were considered as basic control variables. Parental background equaled 1 if at least one parent had completed university-level education by 1980. Additionally, baseline wage differences in 2001 were considered to address potential confounding factors. Models were constructed to include an indicator variable for high education in 2001 while pooling high- and low-educated individuals. Parental smoking status in 1980 (1 = regular daily smoking for at least 1 year; 0 = no regular smoking) was also included as an additional control in robustness analyses due to evidence suggesting intergenerational correlation in smoking behavior.^10^

### Statistical Methods

We employed linear regressions (ordinary least squares, OLS) to analyze labor market differences between smokers and nonsmokers, regressing the logarithm of average annual earnings and the proportion of years employment from 2001 to 2019 on cigarette pack-years while accounting for basic controls. We conducted analyses for all participants, and separately for those with high and low education levels. Coefficient equality between education levels was assessed by computing the point estimates for the coefficients’ linear combinations using the “lincom” command in the Stata software.

To illustrate labor market outcome differences over time, we calculated average earnings and employment rates by smoking status and year for younger (born in the 1970s) and older (born in the 1960s) cohorts. These trajectories were based on raw averages without controls.

To assess potential differences in labor market outcomes between current smokers and individuals who had quit or abstained from smoking in 2001, we employed interaction models, incorporating pack-years, a quitter/abstainer indicator, and their interactions. The interaction term indicates variations in subsequent labor market outcomes between these groups.

## Results

Supplementary Tables S1 and S2 present the descriptive statistics of the sample. In the OLS results (Table 1, column 1), a one-unit increase in pack-years was associated with a 1.8% decrease in earnings (95% confidence interval [CI]: −2.6% to −0.9%, *p* < .001). This suggests that reducing smoking by an amount equivalent to 5 pack-years could lead to a 9% earnings increase (= 5 × 0.018), with effect sizes ranging from 4.5% to 13%. Employment status was not controlled, so the effect size encompasses both wage and employment effects. The quantitative size of the point estimate aligns with an earlier Finnish study, revealing that reducing smoking by 5 pack-years is associated with approximately a 7% increase in income.^11^ Additionally, a one-unit increase in pack-years was linked to a 0.5% decrease in years employed (95% CI: −0.6% to −0.3%, *p* < .001).

**Table 1.** Smoking and labor market outcomes, 2001–2019

		Education		
	(1)	(2)	(3)	(4)
	All	Low education	High education	Equality of coefficients between columns 2 and 3
Panel A: Log of average earnings, 2001–2019				
Pack-years, 2001	−0.018*** [−0.026; −0.009] (*p* < .001)	−0.017*** [−0.029; −0.006] (*p* = .003)	−0.009* [−0.020; 0.001] (*p* = .090)	*p* = .464
Panel B: Proportion of years employed, 2001–2019				
Pack-years, 2001	−0.005*** [−0.006; −0.003] (*p* < .001)	−0.005*** [−0.007; −0.003] (*p* < .001)	−0.003** [−0.006; −0.001] (*p* = .010)	*p* = .435
*N*	1953	1132	821	

Table illustrates OLS regression coefficients, 95% CI (in square brackets), and *p*-values (in parenthesis); additional controls in all models: (indicator for high education), sex, birth cohort (five indicators), an indicator for high family education background, the region of residence in 1980 (three indicators), and the log of earnings in 2001. The equality of the coefficients between columns 2 and 3 was tested by computing the point estimates for linear combinations of the coefficients using the “lincom” command in the Stata software. Statistically significant at

*10%,

**5%,

***1% levels. CI = confidence interval; OLS = ordinary least squares.

The results suggest that the negative relationship between smoking and labor market outcomes was more pronounced among individuals with low education levels (Table 1: columns 2–3). However, the education level difference was not statistically significant (Table 1: column 4). The three categories for education revealed a stronger negative correlation between smoking and labor market outcomes for individuals with only 9 years of comprehensive education compared to those undergoing 12-year intermediate or higher education (Supplementary Table S3). The inclusion of parental smoking indicators did not alter the main results (Supplementary Table S4). Furthermore, shortening the observation period to 2010–2019 accentuated the negative association between smoking and labor market outcomes (Supplementary Table S5).

Supplementary Figure S2 presents the earnings progression over time for smokers and nonsmokers, based on raw averages, highlighting identical earnings trajectories between highly educated smokers and nonsmokers. Initially, there was no earnings disparity between low-educated smokers and nonsmokers. However, over time, low-educated smokers earned less than their nonsmoking counterparts. Supplementary Figure S3 demonstrates similar patterns in employment.

Supplementary Figure S4 reveals a significant earnings difference between smokers and nonsmokers among young cohorts, particularly among the low-educated individuals, with no such distinction among older cohorts. Supplementary Figure S5 depicts a growing employment gap between smokers and nonsmokers among less-educated individuals, which appears to increase with age.

Supplementary Table S6 demonstrates labor market outcome disparities between smokers and individuals who had quit or abstained from smoking in 2001. Among the low-educated population, the negative association between pack-years and employment was primarily attributed to continuing smokers. Those who had quit or abstained from smoking showed no significant negative effects (β = 0.000, *p* = .885, not reported in the table). Moreover, the negative association between pack-years and earnings among less-educated individuals was diminished for those who had quit or abstained from smoking, but this difference was deemed insignificant.

## Discussion

This study unveiled a significant correlation between tobacco smoking and weakened labor market performance in Finland. Two key patterns emerged from the data. First, the earnings gap between smokers and nonsmokers was observed among young cohorts, especially among the low-educated, possibly extending to higher education levels. This disparity was not apparent among older cohorts, suggesting that smoking among younger generations, where it is less prevalent, may negatively affect labor market prospects. Another explanation for the cohort differences is that preexisting influences of smoking on labor market outcomes might reduce the likelihood of detecting subsequent effects among older cohorts. Second, the employment difference between smokers and nonsmokers seemed more prominent among the less educated and appeared to increase with age. Additionally, the adverse association between pack-years and employment among the less educated was driven by ongoing smoking, as this trend was not observed among quitters or abstainers. These findings support the idea that the health impacts of smoking become more evident with age and longer smoking histories, while cessation can reverse several of these effects.^12^ The conclusion that smoking’s adverse effects manifest over time was also supported by the finding that results focusing on the later part of the follow-up-period (2010–2019) showed a stronger negative relationship between smoking and labor market outcomes compared to the results based on the initial time interval (2001–2019).

The adverse employment outcomes associated with smoking seemed more prevalent among individuals with lower education levels, which supports the notion that smoking’s adverse health effects may impact productivity particularly in physically demanding occupations that are more common among less-educated population. Beyond the health implications, smoking could also impact labor market outcomes, potentially affecting employers’ perceptions of productivity. While Finnish law prohibits discrimination, including against smokers, there are few reported cases of discriminatory behavior, making it challenging to assess the extent to which smokers’ disadvantaged positions in the labor market may stem from discrimination.

The study’s strength was in its use of longitudinal register-based employment and earnings data, enabling the longitudinal examination of labor market performance. This is important because the adverse health effects of smoking can accumulate over decades. However, a limitation could arise in the potential clustering of unhealthy behaviors, which could influence our results.^13^ Moreover, unobserved confounders or reverse causality may affect the results. Differences in time preferences,^14^ risk attitudes,^15,16^ or self-control,^17^ for instance, could affect both smoking behavior and earnings, potentially explaining the observed correlations. The number of cigarettes smoked per day was measured in 2001, which may not capture subsequent changes in smoking over time. Potential recall errors in smoking initiation could also lead to measurement error in our pack-year variable, which, if classical, would attenuate our point estimates.

This study’s findings confirm the negative association between smoking and labor market outcomes. A recent study estimated that smoking-related presenteeism, absenteeism, home productivity, and inability to work led to productivity losses worth $184.9 billion in the United States in 2018.^18^ Consequently, the indirect monetary costs of smoking could be economically substantial. It remains unclear whether individuals initiating smoking consider these substantial indirect monetary losses. Previous findings suggest that smokers tend to discount the future more heavily than nonsmokers,^19^ indicating a preference for immediate gains over long-term benefits. Given the prolonged time of manifestation of adverse health and labor market effects of smoking, many individuals may not adequately consider the gradual accumulation of long-term income losses over their professional careers. Designing policy interventions that prompt individuals to consider the indirect health and productivity losses associated with tobacco smoking could help achieve socially optimal outcomes. Several policy tools, including smoking bans, taxes and pricing, and antismoking campaigns, have been implemented to reduce smoking. However, the effectiveness of these policies can vary depending on numerous factors such as age and their impact on smoking prevalence, whether through reducing initiation or increasing cessation rates.^20^ Such heterogeneity may also stem from differences in time preferences and self-control, associated with tobacco smoking. Commitment devices have been proposed as a precommitment tool for individuals with self-control issues and present biases, potentially helping smoking cessation.^21^ However, their effectiveness likely depends on individuals’ awareness of their present bias and self-control challenges. For those unaware of these issues, more paternalistic interventions, such as higher cigarette prices, may be more effective.

## Supplementary Material

Supplementary material is available at *Nicotine and Tobacco Research* online.

ntae296_suppl_Supplementary_Material

## Data Availability

The dataset supporting the conclusions of this article was obtained from the Cardiovascular Risk in Young Finns Study (YFS) and Statistics Finland register data after submission and approval of our study plan by the YFS coordinators. The YFS dataset comprises health-related participant data and their use is therefore restricted under the regulations on professional secrecy (Act on the Openness of Government Activities, 612/1999) and on sensitive personal data (Personal Data Act, 523/1999, implementing the EU data protection directive 95/46/EC). Due to these legal restrictions, the data from this study cannot be stored in public repositories or otherwise made publicly available. However, data access may be permitted on a case-by-case basis upon request only. Data sharing outside the group is done in collaboration with YFS group and requires a data-sharing agreement with YFS representatives and appropriate contracts with Statistics Finland. The linked YFS–FLEED–LPC data can only be used in Statistics Finland remote access system (FIONA). Investigators can submit an expression of interest to the chairman of the publication committee (Prof. Mika Kähönen, Tampere University, Finland).
