# Supplementary material for: Tobacco Smoking in Early Adulthood and Labor Market Performance: The Cardiovascular Risk in Young Finns Study
Source: Nicotine Tob Res. 2025 Jan 14;27(7):1289–93. doi: 10.1093/ntr/ntae296 (PMC12187365; doi:10.1093/ntr/ntae296)
Supplement: ntae296_suppl_Supplementary_Material [file ntae296_suppl_supplementary_material.docx]

**Supplemental Material**

**Supplemental Table 1.** Descriptive Statistics.

|  | Non-smokers  Mean (SD) | Smokers  Mean (SD) | Difference |
| --- | --- | --- | --- |
| **Baseline model** |  |  |  |
| Log of average earnings, 2001-2019 | 7.211  (1.235) | 6.948  (1.602) | 0.263*** |
| Proportion of years employed, 2001-2019 | 0.886  (0.202) | 0.827  (0.266) | 0.059*** |
| Tobacco pack-years, 2001 | 0  (0) | 8.170  (7.704) | -8.170*** |
| High education (proportion), 2001 | 0.522  (0.500) | 0.265  (0.442) | 0.258*** |
| Female (proportion) | 0.574  (0.495) | 0.508  (0.500) | 0.066*** |
| Age, 2001 | 31.552  (4.977) | 31.733  (4.863) | -0.180 |
| Log of earnings, 2001 | 6.138  (2.771) | 5.789  (3.180) | 0.349** |
| High family education background (proportion), 1980 | 0.288  (0.453) | 0.204  (0.403) | 0.084*** |
| Region of residence (proportion), 1980 |  |  |  |
| Southern Finland | 0.150  (0.357) | 0.146  (0.353) | 0.004 |
| Western Finland | 0.368  (0.482) | 0.328  (0.470) | 0.040** |
| Eastern Finland | 0.317  (0.466) | 0.349  (0.477) | -0.032 |
| Northern Finland | 0.165  (0.371) | 0.177  (0.382) | -0.012 |
| N | 1,179 | 774 |  |
| **Labor market outcomes, 2010–2019** | | |  |
| Log of average earnings, 2010-2019 | 7.212  (1.630) | 6.715  (2.466) | 0.497*** |
| Proportion of years employed, 2010-2019 | 0.904  (0.220) | 0.835  (0.303) | 0.069*** |
| N | 1,173 | 765 |  |
| **Parental smoking status** |  |  |  |
| Smoking mother (proportion) | 0.227  (0.419) | 0.354  (0.479) | -0.128*** |
| Smoking Father (proportion) | 0.603  (0.489) | 0.729  (0.445) | -0.125*** |
| N | 1,059 | 649 |  |

Notes: Statistically significant at ** 5 %, *** 1 % levels.

**Supplemental Table 2.** Descriptive statistics: Proportion of smokers among subgroups.

|  | Proportion of smokers in 2001 |
| --- | --- |
| All | 0.292 |
| Cohort born in 1962 | 0.334 |
| Cohort born in 1965 | 0.310 |
| Cohort born in 1968 | 0.277 |
| Cohort born in 1971 | 0.287 |
| Cohort born in 1974 | 0.286 |
| Cohort born in 1977 | 0.254 |
| Female | 0.242 |
| Male | 0.353 |
| Low-educated | 0.394 |
| High-educated | 0.151 |

Notes: N = 1919. Smokers were defined as individuals who smoke at least once a week.

**Supplemental Table 3.** Smoking and labor market outcomes, 2001–2019; education divided into three categories.

|  | (1) | (2) | | (3) |
| --- | --- | --- | --- | --- |
|  | Comprehensive education | Intermediate education | | High education |
| Panel A: Log of average earnings, 2001-2019 | | |  |  |
| Pack years, 2001 | -0.052***  [-0.089; -0.015]  (p = 0.006) | -0.005  [-0.017; -0.007]  (p = 0.423) | | -0.009*  [-0.020; 0.001]  (p = 0.090) |
| Panel B: Proportion of years employed, 2001-2019 | | |  |  |
| Pack years, 2001 | -0.008***  [-0.014; -0.002]  (p = 0.006) | -0.004***  [-0.006; -0.002]  (p < 0.001) | | -0.003**  [-0.006; -0.001]  (p = 0.010) |
| N | 162 | 970 | | 821 |

Notes: Table reports OLS regression coefficients, 95% CI (in square brackets), and p-values (in parenthesis); additional controls in all models: sex, birth cohort (5 indicators), indicator for high family education background, and the region of residence in 1980 (3 indicators), and the log of earnings in 2001. Statistically significant at * 10%, ** 5%, *** 1% levels.

**Supplemental Table 4.** Smoking and labor market outcomes, 2001–2019; models augmented with parental smoking indicators.

|  |  | Education | | |
| --- | --- | --- | --- | --- |
|  | (1) | (2) | (3) | (4) |
|  | All | Low education | High education | Equality of coefficients between columns 2 and 3 |
| Panel A: Log of average earnings, 2001-2019 | | | | |
| Pack-years, 2001 | -0.025***  [-0.035; -0.015]  (p < 0.001) | -0.018***  [-0.031; -0.005]  (p = 0.008) | -0.007  [-0.019; 0.005]  (p = 0.242) | p = 0.333 |
| Panel B: Proportion of years employed, 2001-2019 | | | | |
| Pack-years, 2001 | -0.005***  [-0.006; -0.003]  (p < 0.001) | -0.004***  [-0.006; -0.002]  (p < 0.001) | -0.002*  [-0.005; 0.000]  (p = 0.077) | p = 0.374 |
| N | 1,708 | 959 | 749 |  |

Notes: Table reports OLS regression coefficients, 95% CI (in square brackets), and p-values (in parenthesis); additional controls in all models: (indicator for high education), sex, birth cohort (5 indicators), indicator for high family education background, and the region of residence in 1980 (3 indicators), and the log of earnings in 2001. The parental smoking indicators were measured in 1980 and equaled one if the parent had smoked every day for at least a year (zero otherwise). The equality of the coefficients between columns 2 and 3 was tested by computing the point estimates for linear combinations of the coefficients using the “lincom” command in the Stata software. Statistically significant at * 10%, ** 5%, *** 1% levels.

**Supplemental Table 5.** Smoking and labor market outcomes, 2010–2019.

|  |  | Education | | |
| --- | --- | --- | --- | --- |
|  | (1) | (2) | (3) | (4) |
|  | All | Low education | High education | Equality of coefficients between columns 2 and 3 |
| Panel A: Log of average earnings, 2010-2019 | | | |  |
| Pack years, 2001 | -0.042***  [-0.055; -0.028]  (p < 0.001) | -0.032***  [-0.051; -0.014]  (p < 0.001) | -0.026**  [-0.048; -0.003]  (p = 0.025) | p = 0.738 |
| Panel B: Proportion of years employed, 2010-2019 | | | |  |
| Pack years, 2001 | -0.007***  [-0.008; -0.005]  (p < 0.001) | -0.006***  [-0.008; -0.004]  (p < 0.001) | -0.005***  [-0.008; -0.001]  (p = 0.006) | p = 0.545 |
| N | 1,938 | 1,122 | 816 |  |

Notes: Table reports OLS regression coefficients, 95% CI (in square brackets), and p-values (in parenthesis); additional controls in all models: (indicator for high education), sex, birth cohort (5 indicators), indicator for high family education background, and the region of residence in 1980 (3 indicators), and the log of earnings in 2001. The equality of the coefficients between columns 2 and 3 was tested by computing the point estimates for linear combinations of the coefficients using the “lincom” command in the Stata software. Statistically significant at * 10%, ** 5%, *** 1% levels.

**Supplemental Table 6.** Smoking and labor market outcomes, 2001–2019; models investigating differences between current smokers and those who had quit or abstained from smoking in 2001.

|  |  | Education | |
| --- | --- | --- | --- |
|  | (1) | (2) | (3) |
|  | All | Low education | High education |
| Panel A: Log of average earnings, 2001-2019 | | | |
| Pack years, 2001 | -0.021***  [-0.030; -0.012]  (p < 0.001) | -0.020***  [-0.032; -0.008]  (p = 0.001) | -0.010  [-0.023; 0.002]  (p = 0.097) |
| Pack years × quit/abstained indicator | 0.017  [-0.008; 0.043]  (p = 0.174) | 0.016  [-0.021; 0.053]  (p = 0.403) | 0.004  [-0.022; 0.030]  (p = 0.766) |
| Panel B: Proportion of years employed, 2001-2019 | | | |
| Pack years, 2001 | -0.006***  [-0.007; -0.004]  (p < 0.001) | -0.006***  [-0.008; -0.004]  (p < 0.001) | -0.004**  [-0.006; -0.001]  (p = 0.019) |
| Pack years × quit/abstained indicator | 0.005**  [0.001; 0.009]  (p = 0.023) | 0.007**  [0.001; 0.013]  (p = 0.023) | 0.000  [-0.006; 0.007]  (p = 0.914) |
| N | 1919 | 1112 | 807 |

Notes: Table reports OLS regression coefficients, 95% CI (in square brackets), and p-values (in parenthesis); additional controls in all models: an indicator variable equalling one for those who had quit or abstained from smoking by 2001 (0 otherwise), (indicator for high education), sex, birth cohort (5 indicators), indicator for high family education background, and the region of residence in 1980 (3 indicators), and the log of earnings in 2001. The equality of the coefficients between columns 2 and 3 was tested by computing the point estimates for linear combinations of the coefficients using the “lincom” command in the Stata software. Statistically significant at * 10%, ** 5%, *** 1% levels.

**Supplemental Figure 1.** Flowchart of the study sample.


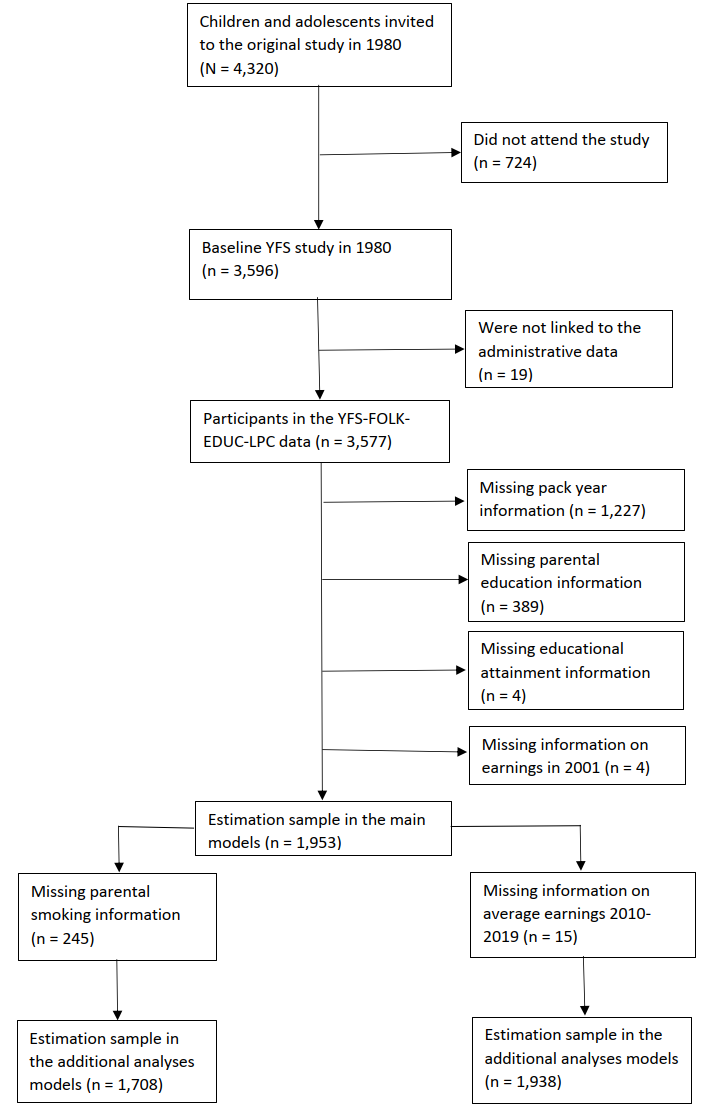


**Supplemental Figure 2.** Average earnings by smoking status, 2001–2019.


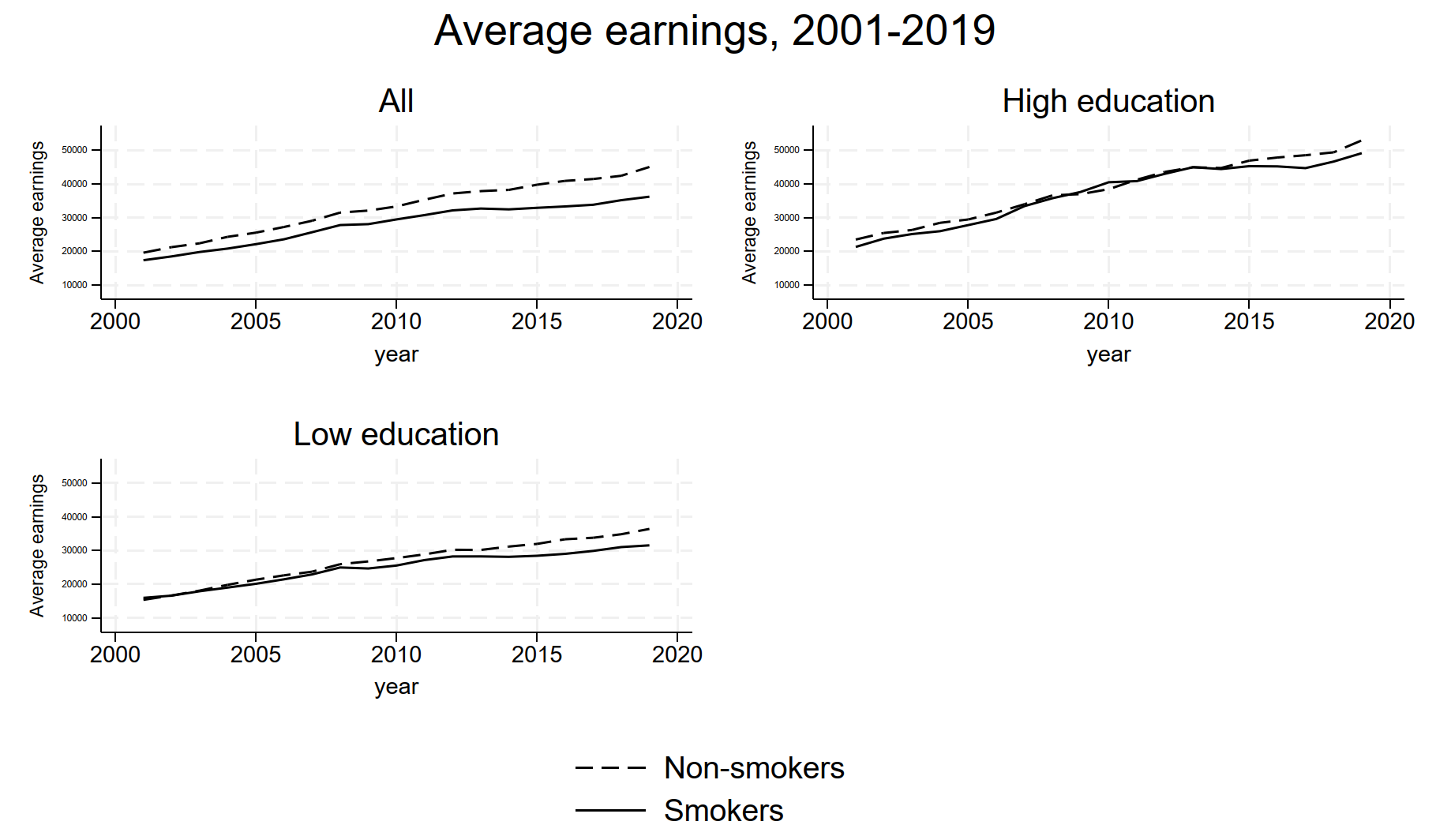


Note: Smokers are defined as individuals with a positive number of pack-years.

**Supplemental Figure 3.** Proportion of years employed, 2001–2019.


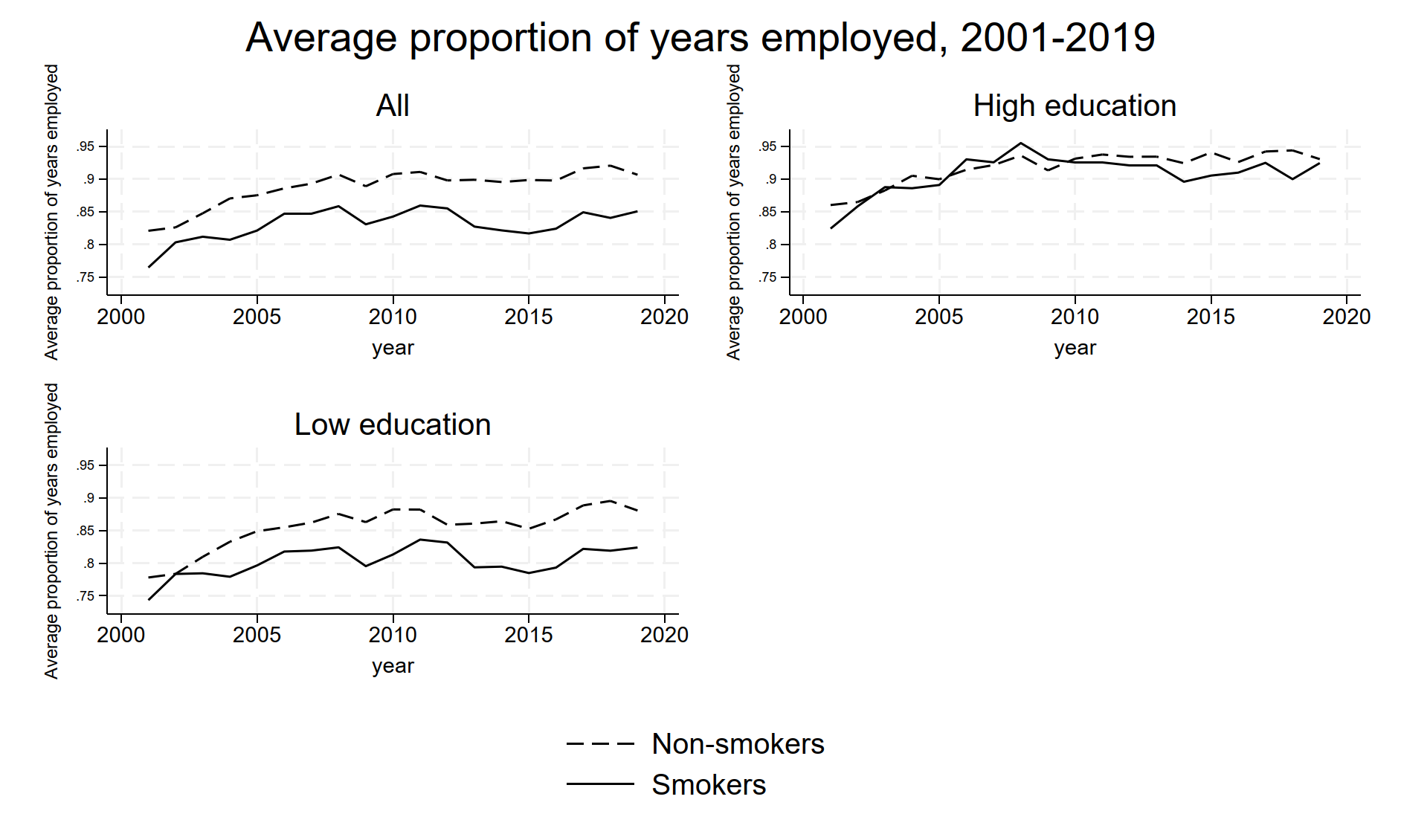


Note: Smokers are defined as individuals with a positive number of pack-years.

**Supplemental Figure 4.** Average earnings by smoking status, 2001–2019; education and cohort stratified results.


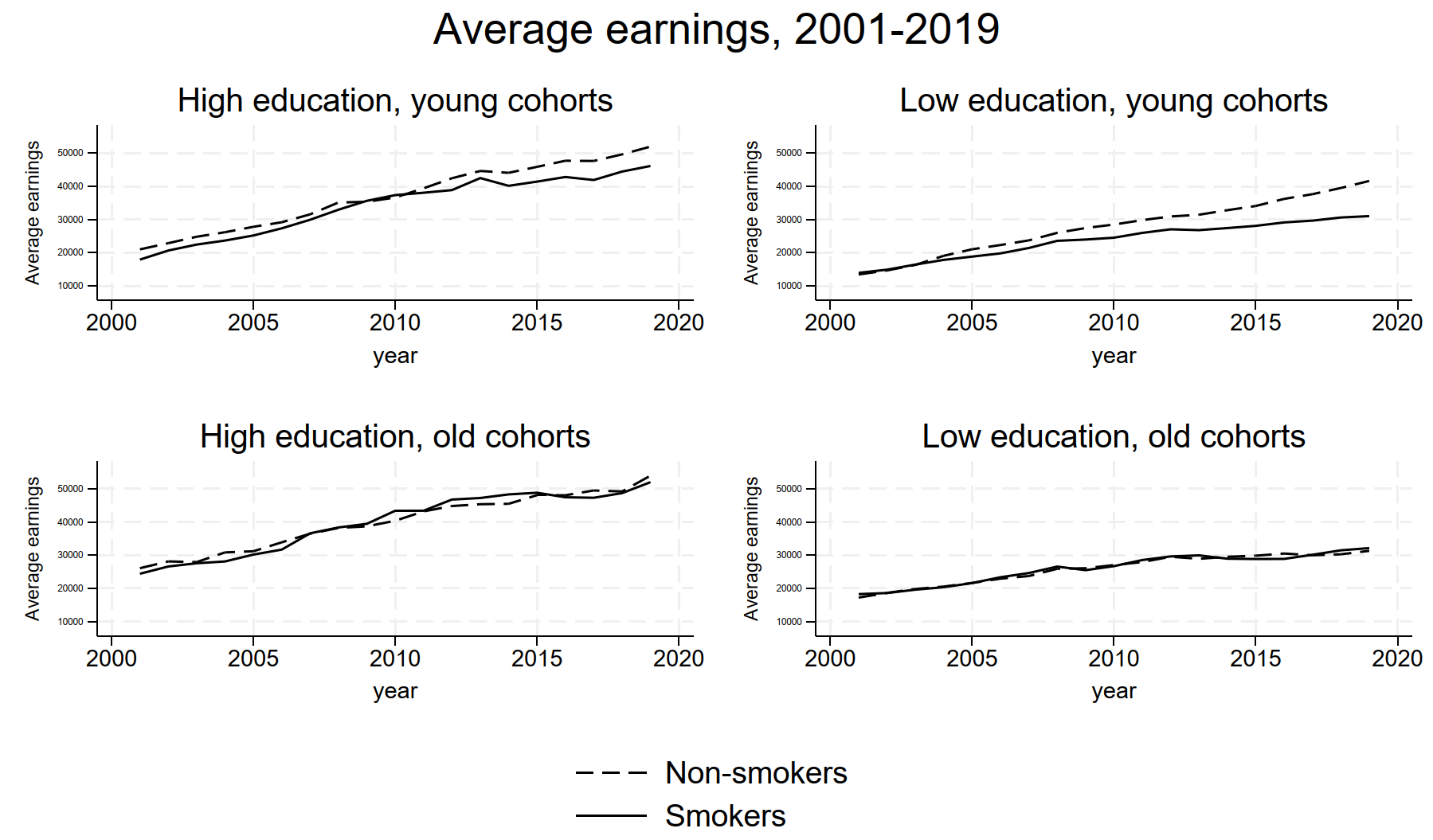


Notes: Smokers are defined as individuals with a positive number of pack-years. “Young cohorts” refer to cohorts born in 1971, 1974, or 1977, whereas “old cohorts” refer to those born in 1962, 1965, or 1968.

**Supplemental Figure 5.** Proportion of years employed by smoking status, 2001–2019; education and cohort stratified results.


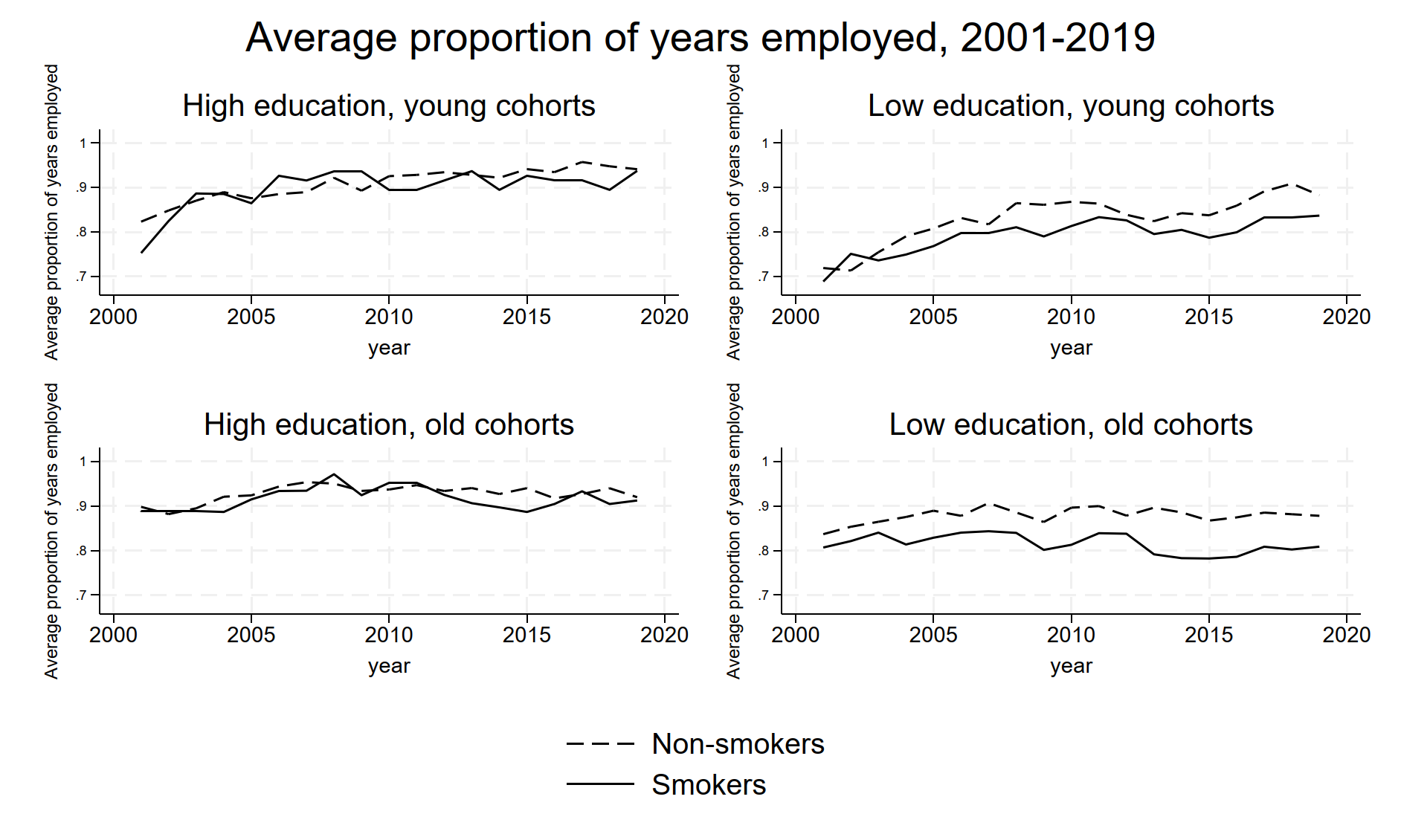


Notes: Smokers are defined as individuals with a positive number of pack-years. “Young cohorts” refer to cohorts born in 1971, 1974, or 1977, whereas “old cohorts” refer to those born in 1962, 1965, or 1968.
